# Lipid Dysregulation in Sebaceous Gland Disorders and the Impact of Sphingolipid Metabolism on Acne Pathogenesis

**DOI:** 10.7759/cureus.82463

**Published:** 2025-04-17

**Authors:** Kaitlyn Miner, Ryan Murphy, Samantha Steiss, McKenzie Burian, Hannah Welp, Alexandra Loperfito, Ashley O'Malley, Radhika Misra, Kelly M Frasier

**Affiliations:** 1 College of Medicine, Kansas City University, Kansas City, USA; 2 College of Osteopathic Medicine, Philadelphia College of Osteopathic Medicine, Philadelphia, USA; 3 College of Osteopathic Medicine, Edward Via College of Osteopathic Medicine, Blacksburg, USA; 4 College of Medicine, University of North Dakota School of Medicine and Health Sciences, Grand Forks, USA; 5 College of Osteopathic Medicine, Lincoln Memorial University DeBusk College of Osteopathic Medicine, Harrogate, USA; 6 College of Osteopathic Medicine, Rocky Vista University College of Osteopathic Medicine, Parker, USA; 7 College of Osteopathic Medicine, Des Moines University, West Des Moines, USA; 8 Department of Dermatology, Northwell Health, New Hyde Park, USA

**Keywords:** acne management, acne vulgaris knowledge, lipid metabolism, sebaceous glands, skin inflammation

## Abstract

Lipid dysregulation within sebaceous glands is a critical factor underlying the pathogenesis of sebaceous gland disorders, particularly acne vulgaris. Sebaceous glands synthesize and secrete a complex mixture of lipids, including triglycerides, wax esters, free fatty acids, and sphingolipids, which contribute to skin barrier function and microbial homeostasis. Dysregulated lipid production, characterized by increased sebum secretion and altered lipid composition, has been implicated in promoting *Cutibacterium acnes* proliferation, hyperkeratinization, and inflammation, key processes in acne pathogenesis. Recent research highlights the pivotal role of sphingolipid metabolism, particularly ceramides, in modulating sebaceous gland activity and skin inflammation. Ceramide deficiencies in acne-prone skin contribute to compromised barrier integrity, increased microbial colonization, and an exaggerated inflammatory response. Furthermore, sphingolipid intermediates such as sphingosine-1-phosphate (S1P) influence sebocyte differentiation, lipid synthesis, and cytokine release, linking sphingolipid metabolism to sebaceous gland homeostasis. Emerging therapeutic strategies targeting sphingolipid pathways offer new opportunities for managing sebaceous gland disorders. Topical and systemic therapies designed to restore ceramide levels and modulate S1P signaling have shown promise in preclinical studies, with potential to reduce sebum production, enhance barrier function, and attenuate inflammation. Additionally, sphingolipid-targeted formulations combined with established treatments, such as retinoids and antimicrobial agents, may enhance therapeutic outcomes while minimizing side effects. The dysregulation of sebaceous lipid metabolism, particularly sphingolipid pathways, plays a central role in acne pathogenesis, and emerging insights into these mechanisms are shaping innovative strategies for the treatment and prevention of sebaceous gland disorders.

## Introduction and background

Acne vulgaris (AV) is a common skin condition characterized by chronic inflammation of pilosebaceous units, which consist of a hair follicle, hair shaft, and sebaceous gland. Regions rich in sebaceous glands are more prone to developing acne, with a characteristic distribution across the face, neck, chest, and back. Acne lesions are classified as either non-inflammatory (comedones) or inflammatory (cystic, nodular, pustular). The resolution of these lesions often leads to scarring or hyperpigmentation [1], further exacerbating the cosmetic implications of AV. The prevalence of acne varies by age and sex, with the highest incidence among adolescents coinciding with the hormonal changes surrounding puberty. While current epidemiologic data suggests a global prevalence of 9.38%, the lifetime prevalence of acne vulgaris is estimated to be 85% [2,3]. Given the significant lifetime burden of this condition, understanding its pathophysiology and developing tailored prevention and treatment strategies can help reduce acne lesions and prevent significant scarring or discoloration.

The development of acne vulgaris is multifactorial, with lipid dysregulation and microbial dysbiosis being major contributors to its pathogenesis. Increased sebum production from sebaceous glands provides a conducive microenvironment for the proliferation of bacteria, such as *Cutibacterium acnes* (*C. acnes*) [4]. *C. acnes*, previously known as *Propionibacterium acnes* (*P. acnes*), exacerbates inflammatory processes and lipid production when in excess. As a result, pro-inflammatory cytokines and oxidized lipids upregulate keratinocyte proliferation, leading to follicular hyperkeratinization and comedone formation [5]. Lipid dysregulation, including deficiencies in sphingolipids, impairs skin integrity and correlates with increased severity of acne lesions [6]. Sphingolipids, particularly ceramide and sphingosine-1-phosphate (S1P), play critical roles in maintaining sebaceous gland homeostasis, suggesting these pathways may serve as therapeutic targets in the treatment of acne vulgaris.

The visibility of acne vulgaris poses significant physical and psychosocial comorbidities. Acne lesions can lead to physical discomfort and contribute to considerable psychosocial distress for individuals affected by the condition [7]. Pressure to abide by beauty standards and desire for social acceptance, especially among adolescents, can adversely impact mental health, contributing to anxiety and depression. Efficacious treatment interventions are shown to improve the morbidity of AV [5], showcasing the importance of further therapeutic developments within this field. This literature review serves to explore the relationship between the dysregulation of sebaceous lipid metabolism and the consequential development of acne vulgaris. Furthermore, this review will examine the role of ceramide-based therapies and modulation of S1P signaling pathways as potential targets for managing AV. Highlighting the role of lipid dysregulation in acne vulgaris pathogenesis may encourage the development of novel therapeutic avenues, thus improving the clinical outcomes and psychosocial distress of individuals impacted by acne lesions.

## Review

Sebaceous gland function 

Sebaceous glands support skin integrity through the production of sebum, a lipid-rich substance essential for maintaining cutaneous homeostasis and protecting the skin from environmental stressors. Located within the dermis, sebaceous glands contribute to nearly 90% of skin surface lipids. Through holocrine activity, sebaceous glands empty sebum onto the hair canal, escaping to the skin surface through a wicking action facilitated by the hair shaft [8]. This process ensures that sebum reaches the epidermis, where it supports skin hydration, thermoregulation, and pH balance. Sebum, the primary excretory product of sebaceous glands, is composed primarily of squalene, wax esters, and triglycerides [9]. These lipid-based components create a fatty fluid that forms a protective barrier across the skin, facilitating thermoregulation by minimizing transepidermal water loss. By emulsifying sweat gland secretions, sebum effectively reduces evaporative cooling, thereby aiding in the retention of body heat [10]. This insulation-like property optimizes cutaneous thermoregulation, particularly in environments where temperature fluctuations pose a challenge to homeostasis. Beyond thermoregulation, the interaction between sebaceous and sweat gland secretions contributes to the maintenance of the skin’s acidic mantle. Free fatty acids produced by sebaceous glands, lactic acid from sweat glands, and resident bacterial flora form an acidic mantle of around 4.5-6.2 pH that helps to control the proliferation of alkaline contaminants [10,11]. An acidic skin pH supports the growth of symbiotic microbiota and promotes skin integrity, both of which can contribute to inflammatory skin conditions when disrupted. 

In addition to their role in maintaining skin homeostasis, emerging literature suggests that sebaceous glands modulate innate immunity within the integument. Sebaceous glands engage in cell-to-cell communication within the skin, interacting with inflammatory cells in the innate immune response through cytokine signaling [12]. Sebocytes engage in dynamic cross-talk with immune cells, secreting pro-inflammatory cytokines that recruit and activate macrophages and T cells in response to microbial and environmental stimuli. Through the production of antimicrobial peptides (AMPs) (small proteins that cause membrane disruption in microbes), sebaceous glands demonstrate bactericidal activity [12], providing an important defense against the overproduction of commensal bacteria. While some AMPs are constitutively secreted, sebaceous glands express receptors that regulate the production of AMPs through proteinase-activated receptor-2 (PAR-2) [12]. Activity at these receptors can alter the antimicrobial profile of sebocytes. This immunomodulatory function positions sebaceous glands as critical components of the skin’s first-line defense, reinforcing their role as both structural and immunological sentinels. 

Despite their essential contributions to skin homeostasis, aberrant sebaceous gland activity is closely linked to the pathogenesis of inflammatory skin disorders, including acne vulgaris. Hormones, age, and diet influence sebaceous gland activity, leading to interpersonal variation in sebum production. Hormone-driven regulation is closely linked to physiological changes that occur throughout various stages of life. Specifically, testosterone and dihydrotestosterone stimulate sebum production and promote the expression and differentiation of sebaceous glands, correlating their activity with peak hormone production periods such as puberty [13,14]. Sebaceous glands are highly concentrated on the face, particularly within the “T-Zone” [15], which is a common site of acne and skin inflammation for many teens and young adults. While males tend to consistently produce sebum from puberty into adulthood, female sebum production declines with menopause [16]. Aside from hormonal influences, dietary choices can also affect sebum production. Diets high in fats and carbohydrates have been shown to increase the activity of sebaceous glands, while diets that restrict calories cause the opposite effect [17]. Specifically, polyunsaturated fatty acids downregulate proinflammatory cascades implicated in acne pathogenesis [17]. Interindividual variability in lipid metabolism and production, however, limits the extent of dietary influence. While regulation of sebaceous glands is crucial in maintaining skin homeostasis, interpersonal variation in hormones, age, and diet can vastly influence inflammatory outcomes.

Acne pathogenesis and lipid dysregulation

Sebaceous gland hyperactivity is a fundamental driver of acne pathogenesis, yet emerging evidence suggests that alterations in sebum composition, in addition to overproduction, play a pivotal role in disease severity among acne patients. These dysregulations in lipid composition include a reduction in linoleic acid (a key protective lipid against comedogenesis) and an increased presence of squalene, which is a highly oxidizable lipid that initiates inflammatory cascades [15]. Upon oxidation, squalene triggers the release of proinflammatory cytokines such as interleukin-6 (IL-6) through lipoxygenase activation within keratinocytes [18]. IL-6 upregulates keratinocyte proliferation [18], contributing to hyperkeratinization when in excess. Systemic modulation of lipoxygenase activity is shown to reduce the number of lesions in individuals with inflammatory acne phenotypes, further demonstrating the role IL-6 plays in lipid dysregulation within sebocytes [18]. Dietary modifications have also been shown to influence sebum lipid profiles, thereby indirectly altering *C. acnes* virulence and inflammation. Specifically, increased triglyceride lipase activity in response to dietary shifts results in elevated free palmitate and oleate levels [19], potent stimulators of inflammatory cascades. Specifically, palmitic acid activates proinflammatory cytokines interleukin-1 beta (IL1β) and tumor necrosis factor-alpha, contributing to sebocyte inflammation and disrupting skin homeostasis [12]. These findings emphasize the intricate interplay between lipid dysregulation and inflammatory responses, highlighting how the multifactorial pathogenesis of acne vulgaris and environmental influences converge to drive disease expression.

In addition to lipid dysregulation, microbial dysbiosis has emerged as a crucial factor in the pathophysiology of acne vulgaris, with alterations in the cutaneous microbiome contributing to the disease's inflammatory phenotype. The proliferation of *C. acnes*, the primary commensal bacteria of the pilosebaceous unit, positively correlates with increased sebum production [20]. This bidirectional relationship between sebocyte hyperactivity and microbial proliferation leads to the occlusion of follicles, promoting further inflammation and the formation of comedones. Comedogenesis, in conjunction with *C. acnes* overgrowth, fosters an anaerobic microenvironment conducive to sustained microbial proliferation [21]. The anaerobic microenvironment generated within these follicles favors the formation of *C. acnes*, which can limit the growth of competing microorganisms. Other commensals exist in lower abundances and exhibit distinct metabolic and immunomodulatory properties that may influence disease progression [22]. Disruptions in the balance between microbes, through the overgrowth of specific strains and depletion of others, can hinder homeostatic immune and inflammatory regulation, as seen in the pathogenesis of acne vulgaris.

While interactions between commensal bacteria play vital roles in maintaining skin homeostasis, changes in bacterial diversity and strain dominance contribute to acne pathogenesis. Recent metagenomic analyses have identified strain-level variations in *C. acnes *that correlate with acne severity [23]. Although *C. acnes* is the dominant microbiota in both acne-affected and unaffected individuals, its pathogenic potential involves strain-specific virulence factors and host-microbe interactions. Specific ribotypes, such as those harboring a G1058C nucleotide substitution in the 16S rDNA sequence, exhibit heightened pathogenicity and are disproportionately represented in acne-prone skin [22]. While reduced microbial diversity, particularly a decline in commensal *C. acnes* subtypes, has been associated with increased acne severity, specific *C. acnes* phylotypes are enriched in healthy skin [22,24]. This suggests a strain-specific contribution to disease susceptibility, with healthy and acne-prone skin demonstrating distinct microbial profiles. The predominance of *C. acnes* phylotype IA1 in seborrheic environments further substantiates a selective advantage for particular strains under conditions of excessive sebum production [24], linking host lipid metabolism with microbial pathogenicity. Taken together, these insights reinforce the paradigm that acne vulgaris is not merely a consequence of sebaceous gland hyperactivity but rather a dynamic interplay between microbial strain heterogeneity, lipid dysregulation, and host inflammatory responses.

Role of sphingolipids in sebaceous gland homeostasis 

Sphingolipids are complex lipids that reside primarily in the plasma membrane, where they play critical roles in cellular structure and function. These molecules share a common sphingolipid backbone but vary based on their attached fatty acid chains. The major classes of sphingolipids include sphingosine, ceramide, sphingosine-1-phosphate (S1P), ceramide-1-phosphate (C1P), and sphingomyelin (SM) [25]. Both ceramides and S1P are essential for maintaining sphingolipid homeostasis, demonstrating opposing effects on cellular processes such as growth, proliferation, and survival [25]. Maintaining sphingolipid homeostasis is critical for preserving the integrity of the skin barrier and preventing various skin disorders. Ceramides are hydrophobic molecules crucial for maintaining the integrity of the epidermal barrier and facilitating the metabolism of membrane components [26]. Ceramides are involved in numerous cell processes, including cellular apoptosis, proliferation, and migration. Ceramides promote apoptosis by altering membrane permeability, which activates mitochondrial caspases that initiate programmed cell death [25]. By triggering apoptosis, ceramides help eliminate damaged or dysfunctional cells, thus ensuring the overall health of the skin. Ceramides contribute to cell signaling through multiple transduction pathways, contributing to their involvement in cellular proliferation. Specifically, C1P activates pathways that upregulate cyclin D1 and c-Myc [25], which regulate cell cycle progression. C1P-activated signal transduction is particularly evident in the proliferation and migration of macrophages [25]. The presence of macrophages within the skin barrier is central to maintaining homeostasis through innate immune responses, including the promotion of tissue repair and modulation of inflammatory responses.

In contrast, sphingolipid-1-phosphate (S1P) promotes cell survival by downregulating caspase activity, thus blocking apoptotic pathways [27]. Sphingosine, a product of the salvage pathway, can be either phosphorylated into S1P by sphingosine kinases (SphK1 and SphK2) or transported to the endoplasmic reticulum (ER). SphK1 exists within the cytosol, while SphK2 is localized to the nucleus and mitochondria. These kinases demonstrate opposing actions on cell survival, with SphK1 promoting survival and mitogenesis [25]. In the ER, sphingosine is recycled to synthesize ceramide and other complex sphingolipids, which are essential for membrane stability and function [27]. This recycling process ensures the continuous renewal of the skin barrier, thereby preserving the skin's protective function. This is particularly important in situations involving tissue repair, such as wound healing and the regulation of inflammation. By preventing excessive cell death and encouraging cell division, S1P ensures that the skin can recover and regenerate effectively in response to injury or environmental stress. Disruptions in sphingolipid homeostasis are implicated in various skin disorders, including acne vulgaris, where imbalances in sphingolipid metabolism contribute to barrier dysfunction and inflammation. Balanced sphingolipid metabolism, particularly the association between ceramides and S1P, is fundamental for preserving epidermal integrity, regulating immune cell activity, and supporting skin regeneration (Table 1).

**Table 1:** Comparison of ceramides and sphingosine-1-phosphate (S1P) in skin function and homeostasis

Feature	Ceramides	Sphingosine-1-phosphate (S1P)
Role in skin barrier	Maintains skin barrier integrity by forming a hydrophobic layer that prevents water loss	Regulates keratinocyte differentiation and promotes re-epithelialization during wound healing
Cellular apoptosis	Promotes apoptosis by altering membrane permeability and activating mitochondrial caspases	Prevents apoptosis by downregulating caspase activity through sphingosine kinase-1
Sebum regulation and acne pathogenesis	Deficiency leads to skin barrier dysfunction, increasing transepidermal water loss and contributing to acne development	Deficiency contributes to microbial dysbiosis, abnormal desquamation, and altered antimicrobial peptide synthesis
Keratinocyte regulation	Supports keratinocyte differentiation through modulation of S1P signaling	Dual effect: promotes keratinocyte differentiation and migration but inhibits excessive proliferation
Therapeutic potential	Used in skincare formulations for barrier repair and hydration and as an adjuvant for pre-existing treatments for acne vulgaris	Targeted as a potential therapeutic for inflammatory skin diseases like psoriasis due to its role in immunomodulation and wound healing

Expression of different S1P receptor subtypes depends on tissue specificity, with S1P1 being the primary subtype found within the skin. S1P signaling is thought to play an integral role in endothelial repair, immune regulation, and keratinocyte activity. Activated platelets release S1P into the cytoplasm, serving as a chemoattractant for endothelial cells [28]. As a result, S1P signaling may contribute to wound healing by promoting the migration and proliferation of endothelial cells. Unlike classic lymphokines, S1P is a weak inducer of T-cell proliferation, and higher concentrations often inhibit chemokine-induced migration of T cells in vitro [28,29], suggesting that S1P plays an immunomodulatory role. High levels of S1P can disrupt inflammatory processes by affecting immune cell trafficking in a concentration-dependent manner. Within the skin, S1P induces keratinocyte differentiation and migration, further supporting its role in the re-epithelialization of wounds [30]. However, S1P demonstrates a paradoxical effect on keratinocyte proliferation. Similar to transforming growth factor-beta (TGFβ), S1P inhibits keratinocyte proliferation while promoting fibroblast matrix production and proliferation [31]. As a result, S1P can prevent hyperkeratinization by suppressing keratinocyte proliferation and promote wound healing by inducing fibroblast matrix proliferation, keratinocyte differentiation, and keratinocyte migration. These diverse functions of S1P, particularly its roles in immune regulation, re-epithelialization, and keratinocyte behavior, emphasize its significance in skin homeostasis and acne pathogenesis.

While increased sebum excretion is a known contributor to acne pathophysiology, emerging literature suggests that a deficiency of S1P plays a significant role in inflammatory pathways and microbial dysbiosis. A sphingolipid deficiency results in follicular hyperkeratosis and abnormal desquamation, both of which contribute to the formation of clogged pores and comedones in acne vulgaris patients [32]. However, conflicting findings exist regarding the systemic role of S1P in acne pathophysiology. A study by Kaya et al. found no significant difference in serum S1P levels between acne patients and controls, though this study was limited by a small sample size [33]. S1P may contribute to acne pathogenesis through other regulatory mechanisms, specifically by influencing antimicrobial defenses. The environment within comedones, which is typically hypoxic and rich in sebum, creates an ideal breeding ground for microbial colonization. This exacerbates inflammation and promotes the growth of acne-related bacteria, such as *C. acnes* [32]. Cathelicidin antimicrobial peptide (CAMP), which is upregulated during epidermal differentiation and concentrated in the stratum corneum, is regulated by S1P [34]. S1P-mediated CAMP synthesis, among other endogenous antimicrobial peptides, is an essential immunomodulatory first-line defense against overproduction of *C. acnes*. Deficiency of S1P may hold clinical significance in the pathogenesis of acne vulgaris through multiple mechanisms, suggesting its modulation as a potential therapeutic target.

Beyond microbial dysbiosis, the pathogenesis of acne vulgaris is strongly associated with abnormalities in the epidermal barrier, particularly in the stratum corneum (SC). Studies indicate that individuals with acne vulgaris often exhibit increased sebum production, elevated transepidermal water loss, and decreased SC conductance, collectively contributing to skin dysfunction [6]. These changes are primarily due to disruptions in the SC that impair the permeability barrier [6], such as a reduction in sphingosine and ceramide levels. The severity of this barrier impairment correlates directly with the clinical severity of acne vulgaris, with lower levels of free sphingosine and total ceramides serving as biomarkers of dysfunction [6]. The interplay between these two molecules is crucial for skin barrier formation and tissue regeneration, ensuring the skin remains resilient and properly functioning under normal and stressful conditions. Deficiencies in either S1P or ceramides may lead to compromised skin barrier function, resulting in increased susceptibility to inflammation, microbial invasion, and excessive sebum production, which are hallmarks of acne vulgaris. Future research investigating targeted modulation of sphingolipid pathways may yield novel therapeutic strategies for acne vulgaris and other inflammatory skin conditions.

Emerging therapeutic strategies targeting sphingolipid pathways 

Restoring ceramide levels in acne-prone skin improves skin barrier function through maintaining skin integrity and reducing susceptibility to inflammation. Ceramide-based therapies are formulated to use existing synthetic pathways to convert precursor molecules into endogenous ceramides [35]. Exogenous ceramides are absorbed into the epidermis of the skin, where they become hydrolyzed by ceramidases into their core components, a sphingoid base and fatty acid. These contribute to a pool of precursor molecules that can be used by existing synthetic pathways to create endogenous ceramides [36]. Ceramides are frequently included as a therapeutic ingredient in skin care products aimed at improving skin barrier function through enhanced skin hydration. Hydroxypalmitoyl sphinganine, for example, is a ceramide precursor found to stimulate the production of ceramides in human skin models and is commonly included as an ingredient in moisturizers and body washes [37]. In restoring the natural lipid composition of skin, hydroxypalmitoyl sphinganine helps reinforce cutaneous integrity within the epidermis. Application of these ceramide precursors is also found to increase keratinocyte differentiation through modulation of S1P signaling, leading to upregulated CAMP production [36]. Exogenous ceramide precursors increase CAMP to different extents, with non-hydroxy ceramide containing dihydrosphingosine (NDS) demonstrating significantly more CAMP than non-hydroxy ceramide containing 4-hydroxy dihydrosphingosine (NP) species [36]. Though NDS and NP are both widely used as topical skincare agents, their therapeutic potential differs as a result of their unique metabolic fates. By promoting a healthier skin microbiome, ceramide-based therapies can target acne vulgaris through reducing inflammatory processes associated with *C. acnes *proliferation. Increasing epidermal ceramide production through the topical application of ceramide precursors is a key therapeutic strategy to improve acne vulgaris, targeting key mechanisms involved in its pathogenesis.

Ceramide-based therapies can be used as an adjuvant to pre-existing treatments for acne vulgaris, which frequently compromise skin barrier integrity. Current therapies for inflammatory acne include benzoyl peroxide, antimicrobials, and retinoids. These treatments, particularly when used in combination, can lead to increased transepidermal water loss, resulting in dehydrated and irritated skin [38]. The incorporation of ceramide-based moisturizers as a complementary therapy to these treatments can prevent further disruption of the skin barrier and alleviate irritation. A double-blind study investigating the use of a ceramide-based moisturizer and skin cleanser in conjunction with adapalene and benzoyl peroxide demonstrated a significant reduction in skin dryness, erythema, and inflammatory lesions compared to a group using only adapalene and benzoyl peroxide [38]. The ceramide therapy not only improved skin irritation associated with adapalene and benzoyl peroxide but also improved the number of acne lesions [38]. The improvement in inflammatory lesions may be attributed to the synergistic effects of ceramide therapy combined with conventional acne treatments. Additionally, incorporating ceramide-based therapy may improve patient adherence to acne regimens, as non-adherence is often a result of skin irritation and dryness [39]. As a result, ceramides improve skin barrier integrity by alleviating irritation and encouraging patient adherence, making them an important therapeutic consideration when prescribing conventional acne treatments.

The topical application of ceramides, however, presents unique challenges in formulation that may impact their utility for clinical consideration. In order to effectively penetrate the skin, ceramides are dissolved at high temperatures and combined with specific solvents that prevent recrystallization during the cooling process [40]. Choosing the wrong solvent can result in undissolved ceramides forming crystals. Recrystallization destabilizes the emulsion, negatively affecting the quality and efficacy of the formula [40]. Novel carrier systems, however, aim to improve the delivery of these lipophilic compounds across the stratum corneum. Ceramide-based microemulsions, nanoparticles, and liposomes have an advantage over conventional carrier systems in their ability to permeate deeper into the skin, delivering larger, more stable molecular structures [41]. These advanced delivery systems may contribute to improved clinical outcomes. Current literature suggests that microparticle preparations of ceramides are associated with improved barrier function in atopic dermatitis [41]; however, evidence supporting the utility of advanced delivery systems in acne vulgaris is lacking. Further research is required to best formulate ceramide-based therapies to improve clinical outcomes in acne vulgaris.

Expanding beyond ceramide-based therapies, modulating S1P signaling presents a novel therapeutic avenue to target the production of inflammatory mediators involved in acne vulgaris pathogenesis. Current FDA-approved S1P receptor modulators include the medications fingolimod, siponimod, ozanimod, and ponesimod. These oral formulations have specific S1P receptor selectivity and are approved to treat multiple sclerosis and ulcerative colitis [42]. Although these medications are not clinically approved to treat cutaneous pathologies, preclinical data demonstrate their utility in treating inflammatory skin disorders. Topical applications of fingolimod have been studied in mouse models for the treatment of atopic dermatitis and psoriasis, showing downregulation of S1P-mediated inflammatory cytokine production [43]. Additionally, oral administration of ponesimod significantly reduced psoriasis severity in a placebo-controlled phase II trial; however, oral formulations of these medications can cause severe systemic side effects, including lymphopenia and bradycardia [43,44]. Extrapolating results from these studies, topical formulations of S1P modulators may mediate the inflammatory properties of acne vulgaris while minimizing the side effects associated with oral dosing. S1P receptor modulators represent a promising avenue for acne vulgaris treatment due to their established roles in regulating inflammation and keratinocyte activity. While direct evidence is still emerging, their potential as targeted therapy for atopic dermatitis and psoriasis suggests a role in treating acne vulgaris.

Challenges and future directions 

While sphingolipids play a vital role in skin barrier function and inflammation, their systemic, off-target effects introduce potential risks that must be carefully considered in acne treatment. While sphingolipid-targeted therapies have shown promise in treating inflammatory dermatologic conditions such as psoriasis [45], the long-term safety of sphingolipid modulation remains unclear, particularly given its systemic effects on immune, cardiovascular, and neurologic function. S1P regulates immune cell trafficking and inflammatory responses, influencing lymphocytes, mast cells, dendritic cells, and macrophages [46]. Modulating the S1P pathway could inadvertently impair immune homeostasis, potentially weakening both innate and adaptive immunity. Additionally, S1P exerts cardioprotective effects by promoting cardiomyocyte survival during ischemic stress, raising concerns that targeting this pathway may heighten susceptibility to cardiovascular events [46]. Similarly, disruptions in ceramide metabolism have been linked to neurodegenerative disorders, such as myoclonic epilepsy [47], further emphasizing the need for cautious therapeutic development. Acne itself is a heterogeneous condition influenced by factors such as sebaceous overactivity, microbial dysbiosis, and immune dysregulation, which may differentially respond to sphingolipid-targeted therapies. A study on phytosphingosine, a ceramide derivative, found reductions in pustules and papules in acne patients, but it did not differentiate between various acne subtypes [48]. Without stratification by disease severity or underlying pathophysiology, treatment efficacy remains uncertain, underscoring the need for larger, more comprehensive clinical investigations.

Beyond pharmacologic intervention, dietary and environmental factors influence sphingolipid metabolism and could serve as adjunctive acne treatment strategies. High-fat diets increase systemic palmitate availability, a precursor to ceramide biosynthesis, suggesting that dietary modifications could theoretically impact inflammatory skin conditions [49]. However, the broader metabolic implications of altering lipid intake remain poorly understood, necessitating further research on nutritional approaches to acne management. Environmental stressors such as ultraviolet radiation, heat exposure, and hypoxia-reperfusion injury also influence ceramide production, presenting another potential avenue for therapeutic modulation [50]. While these factors may provide non-pharmacologic treatment strategies, individual variations in diet, climate, and genetic predisposition complicate standardization. Future research should integrate dermatologic, immunologic, and metabolic perspectives to develop targeted therapies that optimize efficacy while minimizing unintended consequences.

## Conclusions

Acne vulgaris disproportionately affects adolescents, imposing both physical and psychosocial distress. Existing treatments for acne lesions often exacerbate inflammation and irritation, highlighting the need for adjuvant therapies to restore skin integrity and modulate lipid dysregulation. Key factors in acne pathogenesis, such as sebaceous gland overactivity, hyperkeratinization, and microbial dysbiosis, particularly involving *C. acnes*, play a significant role in disease development. Targeting these pathogenic factors by modulating sphingolipid intermediates, such as ceramides and S1P, may provide more effective treatment options for reducing inflammation and improving outcomes in these patients. While pre-clinical trials support the role of sphingolipid modulation in mitigating inflammatory skin disorders, topical and oral applications of S1P receptor modulators for skin conditions are not yet available, necessitating further research into their potential as an innovative therapy for acne vulgaris. Dysregulation of sphingolipid metabolism is a key factor in acne development, and emerging therapeutic modalities targeting these pathways present novel approaches for treating and preventing sebaceous gland disorders like acne vulgaris.
